# Genome-Wide Analysis of the Expansin Gene Superfamily Reveals Grapevine-Specific Structural and Functional Characteristics

**DOI:** 10.1371/journal.pone.0062206

**Published:** 2013-04-16

**Authors:** Silvia Dal Santo, Alessandro Vannozzi, Giovanni Battista Tornielli, Marianna Fasoli, Luca Venturini, Mario Pezzotti, Sara Zenoni

**Affiliations:** 1 Department of Biotechnology, University of Verona, Verona, Italy; 2 Department of Agronomy, Food, Natural Resources, Animals and Environment, University of Padova, Legnaro, Padova, Italy; University of North Carolina at Charlotte, United States of America

## Abstract

**Background:**

Expansins are proteins that loosen plant cell walls in a pH-dependent manner, probably by increasing the relative movement among polymers thus causing irreversible expansion. The expansin superfamily (EXP) comprises four distinct families: expansin A (EXPA), expansin B (EXPB), expansin-like A (EXLA) and expansin-like B (EXLB). There is experimental evidence that EXPA and EXPB proteins are required for cell expansion and developmental processes involving cell wall modification, whereas the exact functions of EXLA and EXLB remain unclear. The complete grapevine (*Vitis vinifera*) genome sequence has allowed the characterization of many gene families, but an exhaustive genome-wide analysis of expansin gene expression has not been attempted thus far.

**Methodology/Principal Findings:**

We identified 29 EXP superfamily genes in the grapevine genome, representing all four EXP families. Members of the same EXP family shared the same exon–intron structure, and phylogenetic analysis confirmed a closer relationship between EXP genes from woody species, i.e. grapevine and poplar (*Populus trichocarpa*), compared to those from *Arabidopsis thaliana* and rice (*Oryza sativa*). We also identified grapevine-specific duplication events involving the EXLB family. Global gene expression analysis confirmed a strong correlation among EXP genes expressed in mature and green/vegetative samples, respectively, as reported for other gene families in the recently-published grapevine gene expression atlas. We also observed the specific co-expression of EXLB genes in woody organs, and the involvement of certain grapevine EXP genes in berry development and post-harvest withering.

**Conclusion:**

Our comprehensive analysis of the grapevine EXP superfamily confirmed and extended current knowledge about the structural and functional characteristics of this gene family, and also identified properties that are currently unique to grapevine expansin genes. Our data provide a model for the functional characterization of grapevine gene families by combining phylogenetic analysis with global gene expression profiling.

## Introduction

Expansins are cell wall-loosening proteins that regulate cell wall expansion and cell enlargement in a pH-dependent manner [1]. The first expansins were found in cucumber (*Cucumis sativus*) [2] but many others have subsequently been identified throughout the plant kingdom [3] and related proteins exist in bacteria, nematodes and mollusks, probably helping to digest plant biomass [4]–[7]. Genome-wide analysis has revealed a large expansin superfamily (EXP) in plants comprising four families [8] defined as expansin A (EXPA), expansin B (EXPB), expansin-like A (EXLA) and expansin-like B (EXLB) according to a standardized nomenclature [9]. Only the largest families (EXPA and EXPB, the latter originally identified as group I pollen allergens) have been shown empirically to loosen cell walls, whereas the expansin-like proteins (EXLA and EXLB) are recognized only by their conserved sequences and their precise functions remain unknown [10], [11].

Expansins are typically 250–275 amino acids in length and comprise a 20-residue N-terminal signal peptide that targets the protein to the cell wall and two major domains, one homologous to glycoside hydrolase family 45 although its activity has yet to be confirmed (domain 1) and another thought to bind cell-wall polysaccharides (domain 2) [8]. The mechanism of action is still largely uncharacterized but may involve the acid-induced disruption of hydrogen bonds linking cellulose and hemicellulose fibers, promoting slippage between the polymers and allowing the cell to absorb water, ultimately leading to cell wall expansion [1], [12]. The shortage of functional information reflects the scarcity of informative structural data, given that the only solved crystal structures represent one bacterial expansin [4] and the pollen-specific EXLB in maize [13]. Although no structural data exist for plant EXPA and EXPB proteins, the developmental and physiological functions of expansins have been studied in many plant species, organs and developmental stages, showing that these proteins are involved in processes such as root and shoot apical meristem development [14], [15], the definition of plant architecture [16], xylem formation [17], abscission [18], [19], seed germination [20], several types of plant–microbe interactions [21]–[23] and fruit softening [24]–[27].

Grapevine (*Vitis* spp., family Vitaceae) is an important perennial woody species because of its economically-valuable role in fruit and wine production, thus it was the first fruit crop selected for genome sequencing [28], [29]. The relatively small grapevine genome (475 Mb) and the absence of recent whole genome duplication (WGD) events means it is also ideal for the genome-wide characterization of gene families. Recent studies have identified many gene families and superfamilies in grapevine, including stilbene synthases [30], dehydrins [31], pathogen-related proteins (PR-10) [32], aldehyde dehydrogenases [33] and transcription factors such as TIFY [34] and NAC [35]. There has been no systematic analysis of grapevine expansins thus far, although the expression profiles of several expansin genes have been studied during berry development [36], [37] and one expansin was identified as minor grape allergen [38]. Recently, certain expansin transcripts have been identified by microarray analysis as markers of berry development [39]–[41].

We screened the V1 annotation of the 12X grapevine genome (PN40024 genotype; http://srs.ebi.ac.uk/) and identified 29 expansin genes representing all four families. This is the first genome-wide study of the grapevine expansin superfamily and we propose a systematic nomenclature based on chromosome position and existing guidelines [9]. We carried out a global analysis of differential expansin gene expression by mining publicly-available microarray data from the recently-published grapevine transcriptome atlas [42] and combining this with data from quantitative real-time RT-PCR experiments. Finally, we focused on the role of expansins in post-harvest withering, a process used in the manufacture of straw wines. Our data provide a basis for the further evolutionary and functional characterization of expansin genes and our exhaustive genome-wide expression analysis has helped to determine the precise role of expansins in grapevine, and thus their potential functions in other fruit crops.

## Results and Discussion

### Identification of expansin genes in grapevine by genome-wide analysis

The genome of the near-homozygous PN40024 genotype of *V. vinifera* cv Pinot Noir was screened for EXP gene sequences. Previously-identified expansin sequences from *Arabidopsis thaliana*, rice (*Oryza sativa*) and poplar (*Populus trichocarpa*) were used as BLASTP queries against the 12X V1 assembly using the JIGSAW software platform [43] at the CRIBI in Padova, Italy (http://genomes.cribi.unipd.it/grape/). This led to the identification of 29 expansin or expansin-like sequences, representing all four expansin families [8]. We identified 24 genes containing the HFD motif which is found only in the EXPA and EXPB families, including 20 in which domain 1 included the characteristic large insertion (α-insertion) and deletion (α-deletion) that is unique to the EXPA family, suggesting that the grapevine genome contains 20 EXPA genes and four EXPB genes (Figure S1). Among the remaining sequences, one was classified as an EXLA gene, based on the absence of the HDF domain and the presence of a characteristic EXLA extension at the C-terminus, and the other four were classified as EXLB genes (Figure S1). All 29 genes were named based on family identity and, within each subgroup, their chromosomal position. The proposed nomenclature for grapevine expansin genes, together with identifiers in the 12X V1 annotation and the length of each open reading frame (ORF), are listed in Table 1. We compared the number of grapevine genes in each expansin family to the other available angiosperm genomes, i.e. Arabidopsis, rice and poplar (Table 2). The EXPA family is the largest, with 20 members in grapevine (*VvEXPA1*–*VvEXPA20*), 26 in Arabidopsis [6], 34 in rice and 27 in poplar [44], [45]. The EXPB family is similar in grapevine, Arabidopsis and poplar, but has undergone a grass-specific expansion in rice [8]. EXLA was the smallest family with only one member in each species, whereas the EXLB family appeared to have expanded specifically in woody species (grapevine and poplar) but not in Arabidopsis and rice.

**Table 1:** Grapevine expansin genes identified in the PN40024 12X V1 prediction.

Proposed nomenclature	PN40024 12Xv1 ID	RefSeq	Coordinates	Strand	ORF (aa)	Gene (bp)
***EXPA***						
*VvEXPA1*	VIT_01s0026g02620	XM_002272843.2	chr1: 12260499..12261641	minus	246	1143
*VvEXPA2*	VIT_02s0025g04700	XM_002275577.1	chr2: 4249304...4250807	minus	264	1504
*VvEXPA3*	VIT_03s0038g04060	XM_002283109.1	chr3: 2934516..2935485	plus	250	970
*VvEXPA4*	VIT_04s0079g00420		chr4: 10905332..10909691	plus	271	4360
*VvEXPA5*	VIT_06s0004g00070		chr6: 173176..174251	plus	247	1076
*VvEXPA6*	VIT_06s0004g04860		chr6: 5816980..5819510	minus	277	2531
*VvEXPA7*	VIT_06s0004g07970		chr6: 8696274..8697795	minus	253	1522
*VvEXPA8*	VIT_07s0005g02310	XM_002273247.2	chr7: 4682986..4683933	plus	256	948
*VvEXPA9*	VIT_07s0005g04850		chr7: 8110218..8111014	minus	240	797
*VvEXPA10*	VIT_08s0058g01200		chr8: 10683123..10683967	minus	249	845
*VvEXPA11*	VIT_08s0007g00440		chr8: 14733277..14734895	minus	258	1619
*VvEXPA12*	VIT_08s0007g04680	XM_002266207.1	chr8: 18624417..18625435	plus	272	1019
*VvEXPA13*	VIT_13s0019g01650	XM_002283322.1	chr13: 3120586..3122103	minus	258	1518
*VvEXPA14*	VIT_13s0067g02930	XM_002280264.2	chr13: 1578700..1580132	plus	252	1433
*VvEXPA15*	VIT_14s0068g00600	XM_002276604.1	chr14: 24335233..24336088	minus	251	856
*VvEXPA16*	VIT_14s0108g01020	XM_002283705.2	chr14: 29649689..29650636	minus	240	948
*VvEXPA17*	VIT_17s0000g06360	XM_002284822.1	chr17: 6888734..6889740	minus	248	983
*VvEXPA18*	VIT_17s0053g00990	XM_002269481.1	chr17: 16772521..16773735	minus	246	1215
*VvEXPA19*	VIT_18s0001g01130	XM_002285855.2	chr18: 1779003..1779933	plus	256	931
*VvEXPA20*	VIT_19s0014g02910		chr19: 3020095..3021255	plus	257	1161
***EXPB***						
*VvEXPB1*	VIT_09s0002g08510	XM_002269154.2	chr9: 9571601..9574374	minus	279	2774
*VvEXPB2*	VIT_12s0059g00190	XM_002267091.2	chr12: 5089208..5091217	plus	259	2010
*VvEXPB3*	VIT_15s0021g02670	XM_002275521.2	chr15: 13673386..13674732	minus	273	1356
*VvEXPB4*	VIT_15s0021g02700	XM_002278523.2	chr15: 13735960..13737451	plus	273	1492
***EXLA***						
*VvEXLA1*	VIT_03s0038g03430	XM_002281815.2	chr3:2487139..2488660	minus	259	1522
***EXLB***						
*VvEXLB1*	VIT_02s0012g00830	XM_002278881.1	chr2:6705411..6707155	plus	255	1745
*VvEXLB2*	VIT_00s0309g00050	XM_002270139.2	chrUn: 22613478..22614823	minus	251	1345
*VvEXLB3*	VIT_00s0309g00090	XM_002269059.2	chrUn: 22639761..22641104	minus	251	1344
*VvEXLB4*	VIT_00s1455g00010	XM_002273860.1	chrUn:39210490..39211840	plus	251	1571

Genes were named based on the expansin family designation and their chromosomal locus. The corresponding identifier in the 12X V1 prediction and open reading frame length are also provided.

**Table 2:** Sizes of the four expansin families in different plants.

	*EXPA*	*EXPB*	*EXLA*	*EXLB*
**Arabidopsis**	26	6	3	1
**Rice**	33	18	4	1
**Poplar**	27	3	2	4
**Grapevine**	20	4	1	4

The number of expansin genes in each family is listed in grapevine, Arabidopsis, poplar and rice.

Experimental evidence has shown that EXPA and EXPB proteins can loosen cell walls [11], [46] whereas EXLA and EXLB have been identified by homology and their activity has not been demonstrated. The similar phylogenetic profile of EXLA and EXLB genes in grapevine and poplar may suggest they perform similar roles in these two perennial woody species.

**Figure 1:**
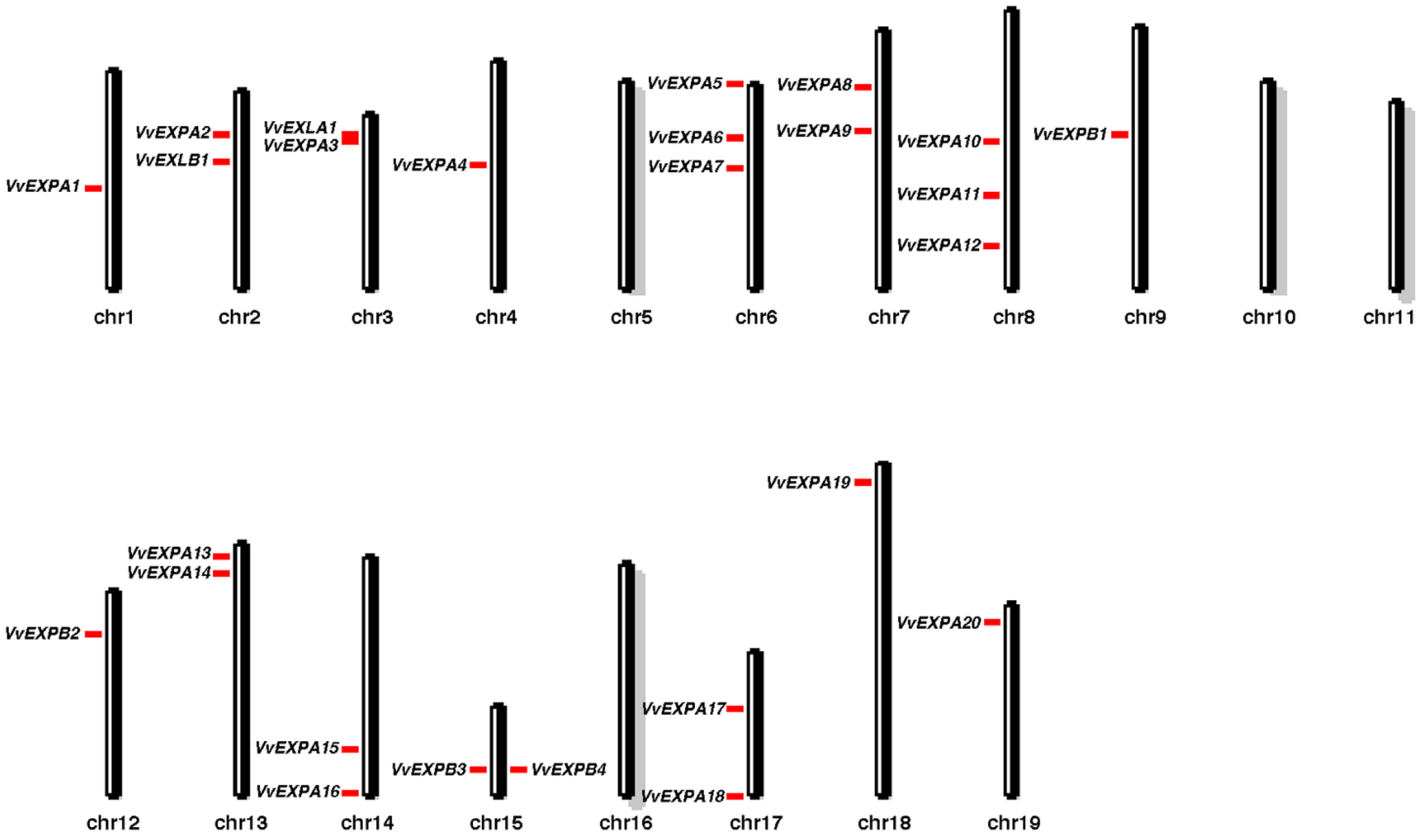
Chromosomal distribution of grapevine expansin genes. Chromosomes bearing no expansin genes are highlighted with gray shading.

The chromosomal distribution of grapevine expansin genes is presented in Figure 1, which shows that paralogs are present on all chromosomes except 5, 10, 11 and 16. The last released grapevine genome assembly did not allow us to assign the positions of three expansin gene models (*VvEXLB2*–*VvEXLB4*) so these could potentially be located on the chromosomes listed above. The distribution of expansin genes is similar in grapevine and poplar, with no more than three expansin genes present on a single chromosome (Figure S2). In contrast, expansin genes tend to be clustered in Arabidopsis and rice, including the presence of 18 expansin genes on rice chromosome 3, reflecting the large number of tandem duplications present in the rice genome (Figure S2).

The expansins do not appear to have undergone such extensive phylogenetic expansion and diversification as other superfamilies, e.g. stilbene synthases, terpene synthases and other genes related to wine characteristics [28]. As proposed for Arabidopsis, the grapevine expansin superfamily may result from massive gene loss following genome duplication.

### Phylogenetic and structural analysis of the grapevine expansin genes

**Figure 2:**
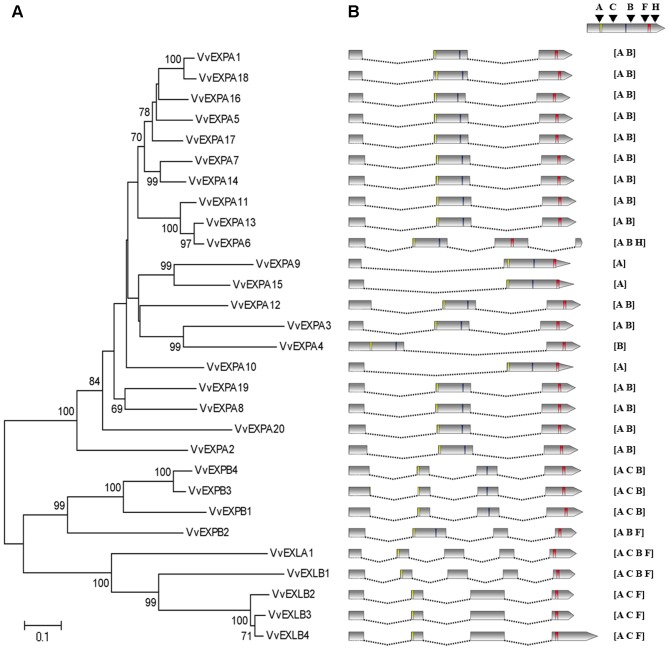
Phylogenetic analysis and exon–intron structures of grapevine expansin genes. (**A**) The deduced amino acid sequences of the grapevine expansins were aligned with MAFFT v6.0 software and the phylogenetic tree was constructed using the neighbor joining method in MEGA v4.0. The reliability of the predicted tree was tested by bootstrapping with 1000 replicates. The percentage of neighbor-joining bootstrap replications (>60%) is shown above each node. (**B**) Exon–intron structures of the grapevine expansin genes. On the left, exons are represented as boxes (drawn to scale) whereas introns are represented as lines (not drawn to scale). Blue, yellow and red boxes in exons indicate the HFD, Box 1 and Box2 motifs, respectively. On the right, the intron structure for each expansin gene is indicated, with positions as previously defined [8]. The intron in position H was defined in the present work.

We constructed a phylogenetic tree including the 29 grapevine expansin proteins based on their deduced amino acid sequences, clustering those in the same families (Figure 2A). To gain more insight into the structural evolution of the expansin gene superfamily, the previously-defined exon–intron boundaries [8] were investigated, revealing that genes from the same family were generally characterized by similar exon–intron structures (Figure 2B). The grapevine EXPA genes generally contained two introns at positions A and B, except *VvEXPA9*, *VvEXPA15* and *VvEXPA10* (which lacked the B intron), *VvEXP4* (which featured an unusually long B intron), and *VvEXPA6*, which contained a third intron at position H, downstream from the BOX 2 site (Figure 2B). The grapevine EXPB genes generally contained three introns at positions A, C and B, except *VvEXPB2* which featured an intron at position F instead of C. Finally, it is noteworthy that the most phylogenetically-divergent EXLB gene (*VvEXLB1*) displayed an exon–intron structure dissimilar to the other *EXLB* genes, but identical to that of the only grapevine EXLA gene (*VvEXLA1*) with four introns at positions A, C, B and F. The highly-conserved sequences and similar exon–intron structures within each family suggest that the families may have undergone independent gene duplications to generate paralogs with partially or completely overlapping functions.

The four expansin gene families in angiosperms were previously subdivided into 17 clades based on a reconstruction of the last common ancestor of monocots and eudicots, which was predicted to contain 10–12 EXPA genes, two EXPB genes, one EXLA gene and two EXLB genes, making 15–17 in total [8]. Each clade would therefore include all of the genes descending from a single gene present in the last common ancestor, constituting a monocot–eudicot orthologous group. We therefore constructed a phylogenetic tree containing all the grapevine expansins and additional Arabidopsis, rice and poplar expansins that represent all the proposed monocot-eudicot clades [8] (Figure S3). We found that all 17 clades were present in the grapevine genome, including EXLB-II and EXPA-IX, which were lost in Arabidopsis but retained in rice and poplar. Based on the minimal ancestral exon–intron structures for each clade [8], we found that most of the grapevine expansins had maintained the ancestral pattern (Figure S3). The exceptions were *VvEXPA12* (clade EXPA-XI), which featured two introns (A and B) instead of a single intron at position B as reported for poplar [45], suggesting the ancestral exon–intron structure of clade EXPA-XI may need to be revised. The EXPA-IX clade includes *VvEXPA4*, which has lost intron A, potentially representing a grapevine-specific intron loss as also reported for some members of clade EXPA-VIII in poplar [45]. Furthermore, an extra intron was found in *VvEXPA6* (clade EXPA-IV) whereas *VvEXPA10* (also clade EXPA-IV) had lost the intron at position B (Figure S3), suggesting either a unique exon–intron structure for certain grapevine expansins or the presence of an independent monocot-eudicot clade which has been lost in Arabidopsis, rice and poplar.

We identified five pairs of grapevine expansin genes apparently reflecting a recent lineage-specific gene duplication event, given their short branches (*VvEXPA1*:*VvEXPA18*, *VvEXPA13*:*VvEXPA6*, *VvEXPA9*:*VvEXPA15*, *VVEXB4*:*VvEXPB3* and *VvEXLB2*:*VvEXLB3*). The duplication of EXLB genes from clade EXLB-I (*VvEXLB2*, *VvEXLB3* and *VvEXLB4*) is particularly remarkable because this appears to be a grapevine-specific event. The majority of grapevine expansin genes appear more closely related to poplar expansins than those from Arabidopsis and rice, which is consistent with the more recent divergence of the grapevine and poplar genomes from a common ancestor than from the lineages leading to monocots and Arabidopsis [28].

Tandem and segmental duplication across different plant lineages has led to the species-dependent expansion of expansin gene families in rice, Arabidopsis and poplar [45]. Grapevine has not undergone a recent whole genome duplication event, but it is unclear whether the haploid grapevine genome (which originated from the contribution of three ancestral genomes) is the product of a true hexaploidization event or successive genome duplications [28]. Recently, it was proposed that the extant triplicate state of the grapevine genome could have arisen from the fusion of two chromosome groups approximate 60 million years ago or by allopolyploidy following eudicot radiation [47]. Therefore, it will be necessary to investigate microsyntenic relationships in order to better clarify the evolution of the grapevine expansins.

### Global analysis of grapevine expansin gene expression

**Figure 3:**
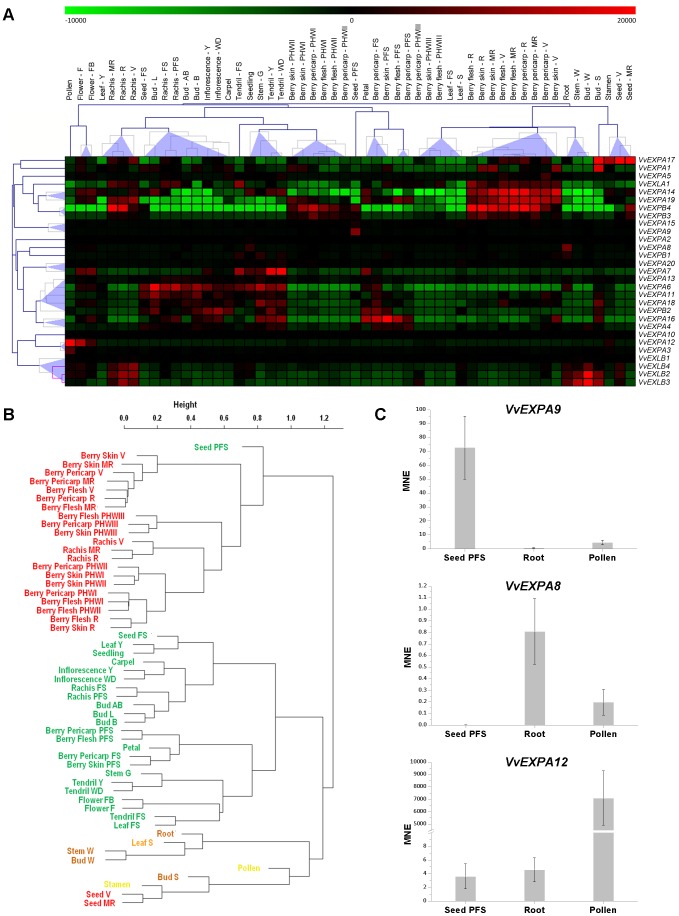
Expression of grapevine expansins in the V. vinifera cv Corvina atlas. (**A**) Expression data were normalized based on the mean expression value of each gene in all tissues/organs. Different organs/tissues are displayed vertically above each column. Gene names are displayed to the right of each row. The color scheme used to represent expression level is red/green: black boxes indicate a low variation in expression, green boxes indicate a fold decrease and red boxes indicate a fold increase with respect to the mean value. Samples and genes were hierarchically clustered based on the average Pearson's distance. Abbreviations indicating developmental stages are listed under Materials and Methods. (**B**) Cluster dendrogram of the 54 grapevine atlas samples, based on the expression profiles within the grapevine expansin superfamily. Pearson's correlation values were converted into distance coefficients to define the height of the dendrogram. (**C**) Organ-specific expansins *VvEXPA8*, *VvEXPA9* and *VvEXPA12* were analyzed by real time RT-PCR in a subset of organs (seed PFS, roots and pollen). Transcripts were normalized to the expression of ubiquitin (*UBQ*). Bars indicate standard error (SE) in three technical replicates.

The recently-published *V. vinifera* cv Corvina gene expression atlas [42] allowed us to characterize the expression of the expansin superfamily in different grapevine organs and developmental stages. We analyzed the fluorescence intensity values of the 29 expansin transcripts, generating a biclustered heat map in which the data were normalized based on the mean center genes/rows adjustment (Figure 3A). The different expansin families shared a common expression profile, with the EXPA, EXPB and EXLA transcripts detected in a wide range of tissues, organs and developmental stages without any particular correlation with the phylogenetic tree. The exception was the EXLB family, whose expression was restricted to organs such as the winter dormant bud (E-L 1), swelling bud (E-L 2), woody stem, root and mature stages of the rachis (V, MR and R).

Pearson's distance correlation analysis was used in a previous study to show that 54 diverse grapevine samples clustered predominantly in relation to temporal dynamics, distinguishing particularly between mature/woody and vegetative/green samples, whereas the organ identity appeared to be less important [42]. We therefore used the same method to cluster the 29 sets of fluorescence data representing the grapevine expansin genes, and the resulting dendrogram was strikingly similar to the one produced in the earlier study (Figure 3B). Temporal-dynamic clustering was preserved in the expansin-specific correlation analysis, highlighting the distinction between mature and vegetative/green samples. These observations indicate that different expansins are predominantly expressed in one or the other grapevine temporal-dynamic cluster and that different organs are characterized by the expression of more than one expansin during its development. Indeed, when grapevine expansin gene expression was analyzed in individual organs, it was still possible to detect the separation between vegetative/green and mature phases (Figure S4). However, the expansin-specific dendrogram also contained a third branch representing woody organs (plus pollen and senescing leaf), which was well-separated from the mature samples. This discrepancy between the expansin dendrogram and the whole-transcriptome atlas reflects the strong co-expression of the EXLB family in woody tissues.

Certain expansin genes were characterized by unique expression profiles in particular tissues, as confirmed by real time RT-PCR (Figure 3C). *VvEXPA9* was expressed uniquely in the seed after fruit setting (Figure 3C, upper panel) and this sample also represents an outlier in the co-expression analysis (Figure 3B), suggesting a highly-specific expansin gene expression profile in seeds at this stage. *VvEXPA8* was expressed predominantly in the root (Figure 3C, middle panel), a phenomenon also observed in other species because expansins are specifically involved in processes such as root hair initiation in Arabidopsis [14] and root elongation in soybean (*Glycine max*) and rice [48], [49]. Finally, *VvEXPA12* was expressed strongly in pollen (Figure 3C, lower panel) and to a lesser extent in the flower at the onset of flowering (10% caps off; Flower - FB) and during flowering (50% caps off; Flower - F), indicating a role in pollen development. No EXPB genes were expressed in grapevine pollen, whereas this family plays a prominent role in monocot pollen development [10]. Our data suggest that only the EXLB family shows tissue-dependent functional specialization in grapevine.

### EXLB genes are expressed in woody tissues

EXLA and EXLB genes contain both domains that characterize the expansin superfamily but the sequence has diverged substantially from the EXPA and EXPB families and no function has yet been established for EXLAs and EXLBs [9].

**Figure 4:**
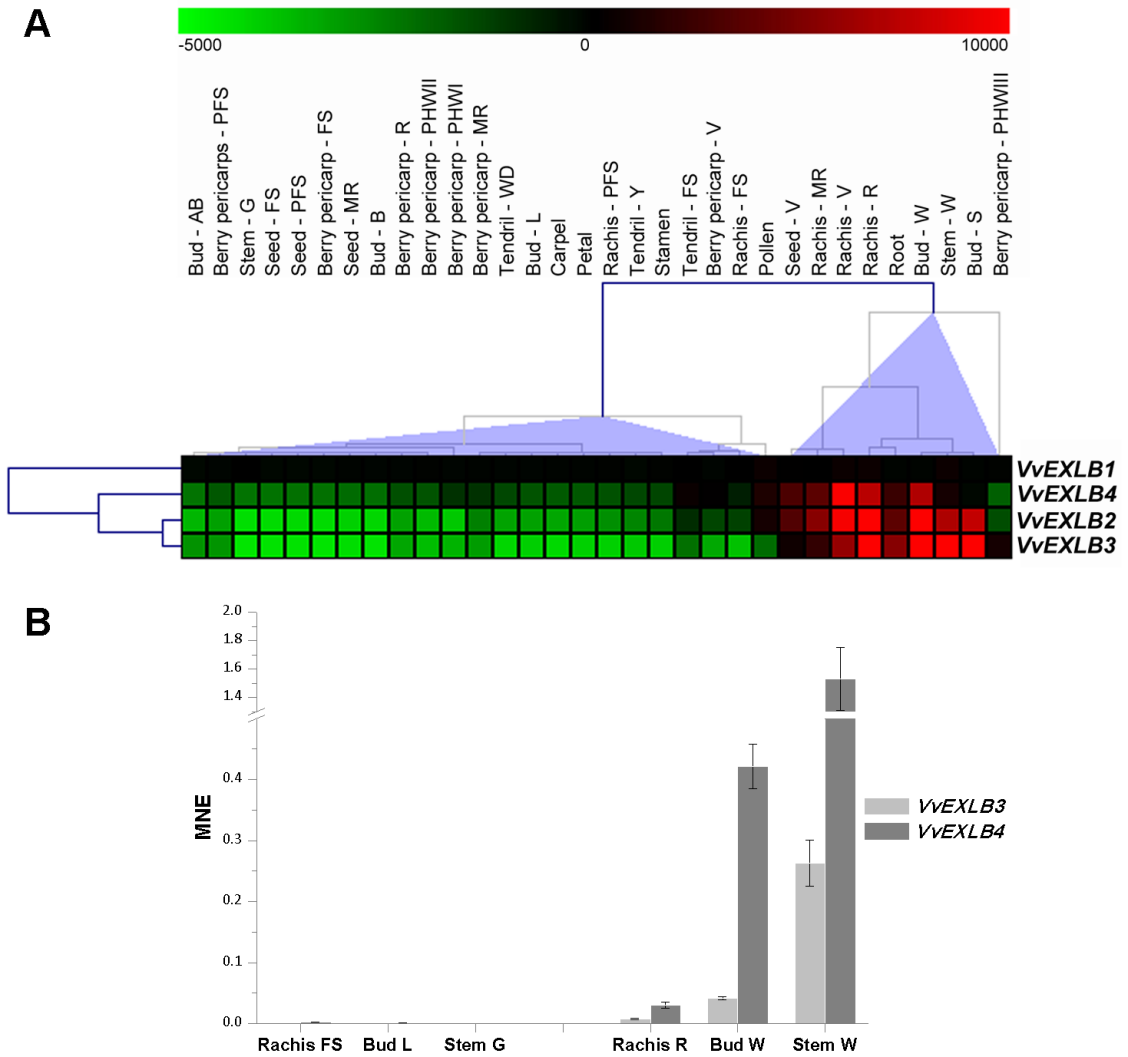
Expression of the grapevine EXLB gene family in woody organs. (**A**) Expression data were normalized based on the mean expression value of each gene in a selected subset of tissues/organs. The color scheme used to represent expression level is red/green: black boxes indicate a low variation in expression, green boxes indicate a fold decrease and red boxes indicate a fold increase with respect to the mean value. Samples were hierarchically clustered based on the average Pearson's distance. (**B**) *VvEXLB3* and *VvEXLB4* were analyzed by real time RT-PCR in rachis, bud and stem both at the green and woody stages to validate data obtained from the expression atlas. Transcripts were normalized to the expression of ubiquitin (*UBQ*). Bars indicate standard error (SE) in three technical replicates.

The EXLB genes showed a unique expression profile in the grapevine gene expression atlas, characterized by little or no expression in the tissues that express EXPA and EXPB genes, such as berries, tendrils, flowers and leaves (Figure 3). Although *VvEXLB1* was not modulated in any of the tissues we analyzed, the remaining EXLB genes (*VvEXLB2*–*4*) were expressed strongly during the mature stages of rachis development (V, MR and R), in winter and swelling buds (bud–W and bud–S), in woody stems (stem–W) and, to a lesser extent, in roots and seeds (Figure 4A). *VvEXLB2* was strongly expressed in all the woody tissues listed above, *VvEXLB3* was induced in the seed and rachis–MR samples, and *VvEXLB4* was induced in woody stems and swelling buds. All these tissues are characterized by various degrees of lignification: by the time berries reach full maturity, the rachis is already brown, dehydrated and lignified, the seed contains 44% lignin (as well as 7% cellulose and 31% hemicellulose) [50], the winter and swelling buds are still enclosed within protective lignified tissues known as perulae, and the woody stem and roots have secondary cell walls.

The microarray expression profiles were validated by real time RT-PCR analysis of individual EXLB genes (*VvEXLB3* and *VvEXLB4*) in rachis, bud and stem tissues (Figure 4B). To emphasize the relationship between EXLB gene expression and secondary cell wall lignification, *VvEXLB3* and *VvEXLB4* transcript accumulation was compared in vegetative/green tissues (green stem, latent bud and rachis at fruit setting) and the same tissues during the mature/woody phase (winter stem, winter bud and rachis at the ripening stage), confirming that *VvEXLB3* and *VvEXLB4* are strongly induced in all woody tissues (Figure 4B).

Expansins are thought to be required for cell wall loosening during primary wall growth [46], which occurs during many different developmental processes in growing and non-growing organs. However, certain expansin genes are expressed specifically in the xylem secondary wall, suggesting a role in secondary wall formation. In Chinese fir (*Cunninghamia lanceolata*), two recently-identified EXPA genes (*ClEXPA1* and *ClEXPA2*) are strongly expressed in wood-forming tissues and preferentially in the cambium [51]. Expansins are also expressed during primary xylem formation in zinnia (*Zinnea elegans*) [52]. In hybrid aspen (*Populus* spp.), three EXPA genes (*PttEXPA1*, *PttEXPA2* and *PttEXPA8*) and one EXPB gene (*PttEXPB1*) are expressed in the cambium and adjacent radial zone of mature stem tissues, supporting a role for expansins in secondary cell wall formation [17]. A recent transcriptome-wide analysis of the transition from primary to secondary stem development in *Populus trichocarpa* identified four EXPA genes and one EXLA gene whose expression profiles were linked to secondary growth [53]. The lack of functional data for EXLB genes means their precise role is difficult to establish. However, an intriguing observation is that the expansin superfamily in the moss *Physcomitrella patens* is similar in number to that in Arabidopsis, but lacks EXLA and EXLB genes [54]. Generally, bryophytes are land plants that do not have vascular tissues and do not contain lignin. This observation supports a relationship between the presence/action of expansin-like sequences such as EXLA and EXLB and the range of processes linked to lignification. Furthermore, a recent investigation of vascular overgrowth in Arabidopsis following treatment with an auxin transport inhibitor identified two EXLB sequences induced in vascular tissues and hydathodes [55]. Taken together, these data clearly indicate that EXLA and EXLB are specifically involved in secondary wall formation and support our proposal that at least *VvEXLB2*, *VvEXLB3* and *VvEXLB4* play specific functional roles in grapevine woody tissues and organs.

### Berry growth and ripening require the activities of different expansin genes

The economical importance of grapevine depends largely on the quality of its berries, which undergo a long process of development and ripening involving a series of dramatic physical and biochemical changes. These include several cell wall structural modifications which are required for cell expansion and softening [56]. During berry development, there are two distinct periods of rapid growth [57]. The first growth phase involves an increase in cell number and volume, whereas the second involves an increase in volume only and is also characterized by softening.

**Figure 5:**
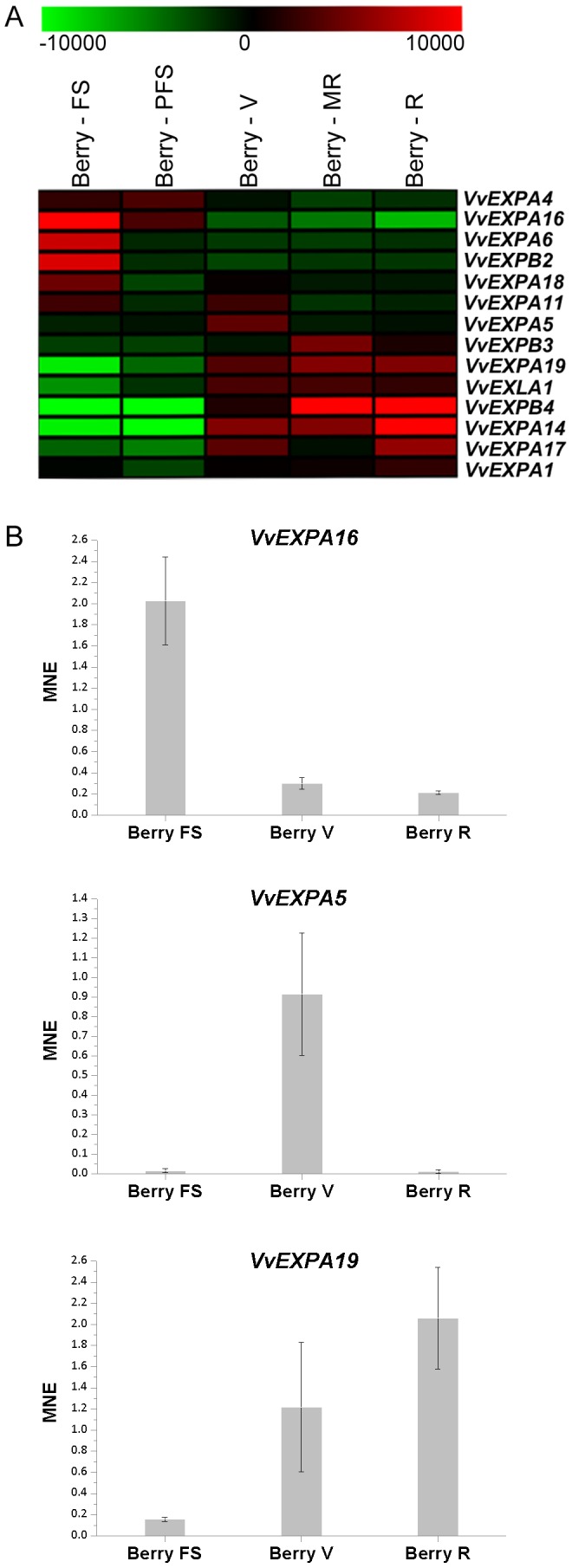
Grapevine expansin gene expression during berry pericarp growth and ripening. Expression data were restricted to berry development from fruit set to ripening. Genes were variance-filtered eliminating 50% of the expansin genes showing low variation in expression among the different stages. (**B**) The expression of selected expansin genes, specifically expressed in different berry developmental stages, was validated by real time RT-PCR. Transcripts were normalized to the expression of ubiquitin (*UBQ*). Bars indicate standard error (SE) in three technical replicates.

We investigated the role of expansins in berry development by focusing on the transcriptional changes during fruit-set (FS) and post-fruit set (PFS) in the first growth phase, and during veraison (V), mid-ripening (MR) and ripening (R) in the second growth phase. The expression profiles of 14 grapevine expansin genes that show significant modulation of expression in at least one of the five samples (encompassing all expansin families except EXLB) are shown in Figure 5A. We identified 10 EXPA genes in clades EXPA-I and EXPA-II, suggesting these are particularly relevant during fruit development, as well as three EXPB genes with berry-specific expression (*VvEXPB2*–*4*) and also *VvEXLA1*, whose global expression profile suggests a role in berry ripening and rachis development (Figure 3A). These expression profiles divide the samples in two clear groups: berries before veraison and berries during and after veraison (Figure 5A). This is consistent with the contrast between vegetative/green and mature samples on the basis of expansin expression profiles (Figure 3B).

The expansins expressed before veraison were *VvEXPA4* and *VvEXPA16* (validated by real-time RT-PCR as shown in Figure 5B), as well as *VvEXPA6*, *VvEXPB2* and *VvEXPA18*. Interestingly, *VvEXPA11* was expressed in the FS and V samples and only at low levels, suggesting a marginal involvement in berry development (Figure 5A). We identified eight expansin genes whose expression was upregulated during the second growth phase. The majority of these (including *VvEXPA19*, *VvEXLA1*, *VvEXPB4*, *VvEXPA14* and *VvEXPA17*) had similar expression profiles albeit with different degrees of induction. *VvEXPB3* and *VvEXPA1* showed single expression peaks in the MR and R samples, respectively, whereas *VvEXPA5* was expressed only at veraison, suggesting a specific role during the second phase of berry growth. The expression profiles of *VvEXPA19* and *VvEXPA5* were also validated by RT-PCR (Figure 5B).

We noted that some members of the expansin superfamily were expressed specifically either in the berry skin or pulp. For example, *VvEXPA1* was expressed only in the skin whereas *VvEXPA17* was found predominantly in the flesh (Figure 3A). This suggests that common expansin substrates are modified specifically within each tissue and that different expansins contribute to the fine tuning of cell wall metabolism in the berry flesh and skin.

Previous studies have shown that some cell wall-modifying enzymes, including expansins, are expressed in a bimodal fashion in which high expression levels coincide with the two periods of rapid berry growth and cell expansion [37]. Transcriptome analysis has also demonstrated that the abundance of most expansin transcripts increases or decreases steadily during berry development [39], [58] although some show characteristic expression profiles matching the second phase of fruit enlargement and softening [39], [59], [60]. One expansin transcript was also shown to be a potential negative marker (i.e. conspicuous by its absence) of the pre-ripening/ripening phases [41]. Expansin genes expressed during the first rapid period of berry growth period may be required for primary cell wall loosening and the incorporation of new cell wall material, whereas those expressed after veraison may regulate contact between cell wall polymers and the enzymes that degrade them, facilitating cell wall disassembly and fruit softening [36], [37]. The classification of grapevine expansin genes involved in the modulation of berry cell wall metabolism, which is a key molecular event in berry development and ripening, could be used to improve berry quality traits and thus the economic value of grapevine.

### Grapevine expansins are involved in water-deficit stress responses

A link has been established between expansins and water-deficit stress, as demonstrated by the induction of several expansin genes by dehydration stress [61], [62] (as well as the induction of others by heat, shading, anoxia and pathogens [63]–[67]) and the ability of expansin transgenes to confer drought tolerance, e.g. in transgenic tobacco (*Nicotiana tabacum*) expressing *TaEXPB23* [68], [69] and in transgenic rose (*Rosa* spp.) expressing *RhEXPA4* [70]. In this context, expansins may regulate adaptive changes in the cell wall structure to increase flexibility and thus prevent damage caused by the folding of rigid cell walls as the cell volume is reduced.

**Figure 6:**
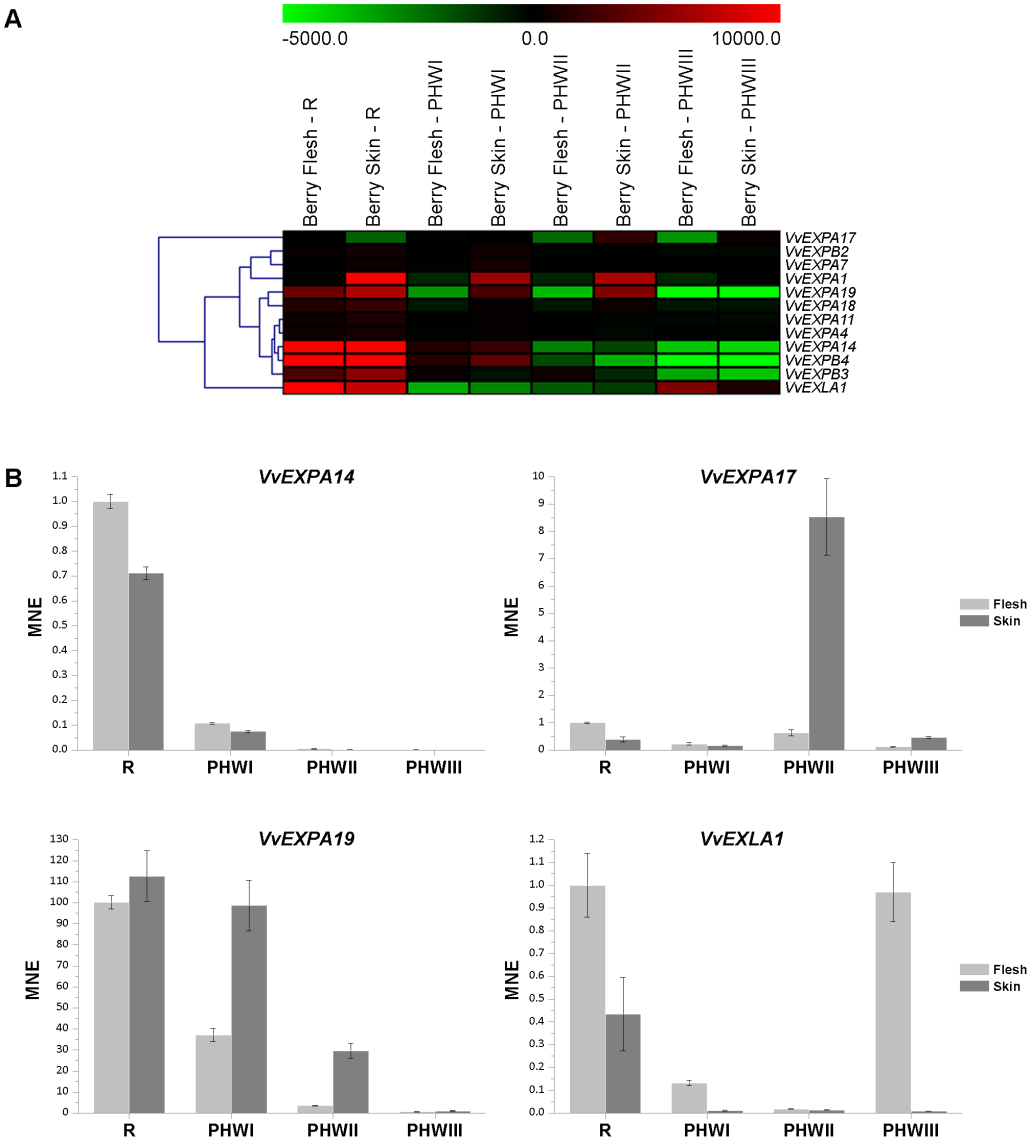
Grapevine expansin gene expression in berry tissues during post-harvest withering process. Expression data from the ripening stage were included for comparison. Genes were variance-filtered eliminating 50% of the expansin genes showing low variation between samples. Selected expansin genes (*VvEXLA1*, *VvEXPA19*, *VvEXPA17* and *VvEXPA14*) were validated by real time RT-PCR. Transcripts were normalized to the expression of ubiquitin (*UBQ*). Bars indicate standard error (SE) in three technical replicates.

Samples representing controlled biotic and/or abiotic stress were not included in the grapevine gene expression atlas, but three stages of post-harvest berry withering were included [42]. This is a controlled form of post-harvest dehydration which is used to improve certain berry quality traits for the production of premium wines. During withering, the berries undergo several chemical and physical changes representing metabolic processes such as the modification of cell wall polymers in the skin [71], [72]. A recent transcriptomic survey of withering in Raboso Piave berry skins [73] showed that *VvEXPA1* transcript levels declined during this period. We therefore used the grapevine expression atlas data to investigate the role of grapevine expansins during post-harvest withering (Figure 6A). In Corvina berries, 12 expansin genes were differentially modulated during withering and we analyzed their expression profiles separately in the flesh and skin. *VvEXPA14*, *VvEXPB3* and *VvEXPB4* were expressed in both tissues and were downregulated during withering. We validated the *VvEXPA14* expression profile by real-time RT PCR (Figure 6B). The other expansins showed a more complex expression profile, e.g. *VvEXPA17* was upregulated at the second withering time point (PHWII) specifically in the berry skin, as confirmed by real-time RT PCR (Figure 6B). *VvEXPA19* and *VvEXPA1* were modulated from the onset of the stress treatment (PHWI) and their expression was also skin-specific (Figure 6A, 6B). Interestingly, *VvEXLA1* expression was induced only in the berry flesh at the last stage of the dehydration process, when weight loss approached 30% (Figure 6A, 6B). The expansins that were induced during withering represent candidate dehydration stress response markers. The coordinated activity of pectin methylesterase (PME) and polygalacturonase (PG) has recently been shown to degrade pectins in Corvina berry skin [74]. Therefore, the skin-specific expansins induced during dehydration (e.g. *VvEXPA19* and *VvEXPA1*) may be involved in pectin metabolism, probably by regulating contact between pectins and pectin-degrading enzymes. Our data suggest that expansins contribute to post-harvest dehydration stress responses by regulating cell wall metabolism in berries.

## Conclusion

We carried out a genome-wide survey of the expansin superfamily in grapevine to gain insight into the chromosomal organization, phylogenetic relationships and expression profiles of these genes. The grapevine genome contains 20 EXPA genes, four EXPB genes, one EXLA gene and four EXLB genes, representing all 17 monocot-eudicot clades proposed for Arabidopsis and rice as well as a potential novel clade not identified thus far in other species, represented by *VvEXPA10*. Most of the grapevine expansin genes retained the ancestral exon–intron structure, although some showed unique structures that may be grapevine specific. A comparison of the grapevine, Arabidopsis, rice and poplar expansin genes revealed that grapevine and poplar expansins were the most closely related and that the grapevine expansins had not undergone significant phylogenetic expansion. However, integrated phylogenetic characterization and gene colinearity analysis within and between plant genomes will be necessary to understand the evolution of the grapevine expansin superfamily in more detail.

Genome-wide gene expression analysis indicated that members of the same expansin family did not necessarily share common expression profiles although most grapevine EXLB genes were expressed strongly in woody tissues and organs, suggesting a specific role in the formation of secondary cell walls. Correlative analysis of expansin gene expression profiles suggested temporal-dynamic clustering in which mature samples and vegetative/green samples were clearly separated.

We also identified the expansins whose expression was significantly modulated during berry development, particularly in the context of the two distinctive phases of berry growth. All expansin families except EXLB were found to be involved in berry development, supporting previous reports that cell wall modification is a critical component of fruit growth and maturation.

Finally, we investigated expansin expression during post-harvest withering, which showed that some expansins are induced by dehydration, indicating a potential role in the control of adaptive cell wall modification under stress. Our data lay the groundwork for the further structural, functional and phylogenetic characterization of expansin genes and their developmental role in commercially valuable fruit crops.

## Materials and Methods

### Database searches, analysis of gene structure and chromosome mapping

Previously-identified Arabidopsis and rice expansin protein sequences (AtEXPA1, AtEXPB1, ATEXLA1, ATEXLB1, OsEXPA1, OsEXPA24, OsEXPB2, OsEXPB15, OsEXLA1 and OsEXLB1) were used as BLAST queries against the Genoscope Grape Genome Database (http://www.genoscope.cns.fr) representing the 8.4X and 12X V0 assemblies of the PN40024 genotype. The search was extended by matching expansin genes and their chromosomal coordinates against an uploaded version of the PN40024 12X assembly V1 (http://genomes.cribi.unipd.it/), which merges the 12 V0 prediction based on GAZE software [75] and a gene prediction performed with JIGSAW software [43] at the CRIBI Genomics in Padova, Italy. Sequences were processed using Vector NTI v9 (Invitrogen) and the gene structure was deduced from the Genoscope and CRIBI annotations or by manual editing based on comparisons with corresponding expressed sequence tags and deduced protein sequences from paralogous expansin genes. The exon–intron structures were presented using the online tool FancyGene [76]. The chromosomal loci were inferred from the genome browsers listed above and rendered using NCBI MapViewer (http://www.ncbi.nlm.nih.gov/).

### Phylogenetic analysis

Multiple alignments of the 29 deduced expansin protein sequences from grapevine, together with 16 from rice, 21 from poplar and 21 from Arabidopsis, were created using the E-INSI tool in the MAFFT v6.0 software suite, which takes into account the possibility of large gaps in the alignments [77]. An unrooted phylogenetic tree was generated using the neighbor joining method [78] in MEGA v5.0 [79]. The reliability of the tree was tested by bootstrapping with 1000 replicates, and the resulting tree was edited and modified with MEGA v5.0 and Treedyn software (http://treedyn.org). An additional tree was produced with the same tools considering only the grapevine expansin gene sequences predicted from the 12X V1 assembly of the PN40024 genotype.

### Analysis of the *V. vinifera* cv Corvina expression atlas

The expression profiles of grapevine expansin genes were analyzed in the global *V. vinifera* cv Corvina (clone 48) gene expression atlas of different organs at various developmental stages [42]. The gene expression microarray data were obtained by hybridization to a NimbleGen microarray 090818 Vitis exp HX12 (Roche, NimbleGen), which contains probes targeting 29,549 predicted grapevine genes (http://ddlab.sci.univr.it/FunctionalGenomics/), representing ∼98.6% of the genes predicted from the V1 annotation of the 12× grapevine genome sequence (http://srs.ebi.ac.uk/) and 19,091 random probes as negative controls. The Corvina expression atlas comprises the following tissues: *in vitro* roots, green stem, buds after bud burst (rosette of leaf tips visible), young leaves (leaves collected from shoots with only five leaves), senescing leaves (leaves at the beginning of leaf-fall), berry rachis (from fruit-set to ripening), flowers (50% cap-fall). In particular, berry pericarp was collected at five developmental stages: the first stage (15 days after flowering (daf); E–L 29) corresponds to the fruit set (FS), when young berries are enlarging (>3 mm diameter); the second stage (35 daf; E–L 32) is the post-fruit set stage (PFS), when berries (>7 mm diameter) start touching; the third stage (70 daf; E–L 35) is the veraison (V), when berries begin to change colour and enlarge (10.4° Brix); the fourth stage (84 daf; E–L 36) corresponds to the mid-ripening (MR) stage (15.5° Brix); and the final stage (115 daf; E–L 38) represents complete ripening (R) (20.0° Brix). The atlas also contains a set of expression data obtained from berries that have undergone post-harvesting withering for 1–3 months after harvest: At the first withering stage (WI), berry weight was 76.4% the ripe value and the sugar content was 24.5° Brix. The second stage (WII) was characterized by 69.7% berry weight and 25.9° Brix, and the last stage (WIII) was characterized by 67.3% berry weight and 26.7° Brix. The expression data were analyzed using T-MeV v4.81 [80], normalized based on the mean center genes/rows adjustment and (in the analysis of individual organs) were also variance filtered with a 50% cut-off. Abbreviations after organ names indicate the developmental stage. FS, fruit set; PFS, post fruit set; V, veraison; MR, mid-ripening; R, ripening; PHWI, post-harvest withering (1^st^ month); PHWII, post-harvest withering (2^nd^ month); PHWIII, post-harvest withering (3^rd^ month), Bud - L, latent bud; Bud - W, winter bud; Bud - S, bud swell; Bud - B, bud burst; Bud - AB, bud after burst; Inflorescence - Y, young inflorescence with single flowers separated; Inflorescence - WD, well developed inflorescence; Flower - FB, flowering begins; Flower - F, flowering; Tendril - Y, young tendril; Tendril - WD, well developed tendril; Tendril - FS, mature tendril; Leaf - Y, young leaf; Leaf - FS, mature leaf; Leaf - S, senescing leaf; Stem - G, green stem; Stem - W, woody stem.

### Correlation analysis

A correlation matrix was prepared using R software and Pearson's correlation coefficient as the statistical metric to compare the values of the 29 members of grapevine expansin genes in all 54 samples [42], using the average expression value of the three biological replicates. Correlation values were converted into distance coefficients to define the height scale of the dendrogram.

### RNA extraction, cDNA synthesis and real-time RT-PCR

Total RNA was isolated using the Spectrum™ Total RNA kit (SIGMA-Aldrich, St. Louis, MO), with sample-specific modifications of the extraction protocol, as previously described [42]. First strand cDNA was synthesized using 1 µg of total RNA as the template and the Improm-II™ Reverse Transcription system (Promega), according to the manufacturer's instructions. The total RNA was derived from *Vitis vinifera* cv. Corvina organs harvested at the same phenological phase as described in the grapevine gene expression atlas [42], and all RNA samples were first treated with DNase I (Promega). Gene-specific primers were targeted, when possible, to the 3′-UTR, using ubiquitin (VIT_16s0098g01190) as the reference gene (Table S1). Primers and cDNA were mixed with the Power SYBR® Green PCR Master Mix (Applied Biosystems, Foster City, CA, USA) and the reaction was carried out on a Stratagene MX 3000 P™ QPCR System (Agilent, Technologies, CA) using the following cycling conditions: 95°C hold for 10 min followed by 45 cycles at 95°C for 30 s, 55°C for 30 s and 72°C for 20 s. Nonspecific PCR products were identified by inspecting dissociation curves. In addition, the specificity of EXLB PCR products was verified by cloning the relative amplicons in the pGem®-T Easy Vector (Promega), sequencing and aligning them onto the reference genome. Amplification efficiency was calculated from raw data using LingRegPCR software [81]. Standard error (SE) values were calculated as described [82].

## Supporting Information

Figure S1 Alignment of grapevine expansin protein sequences. Alignment of all grapevine expansin deduced protein sequences representative of the VvEXPA, VvEXPB, VvEXLA and VvEXLB families. The alignment was created using MAFFT v6.0 and edited with GeneDoc. The family-specific conserved motifs and active sites are indicated.
(TIF)

Figure S2 Gene frequency per chromosome of expansins in Arabidopsis, poplar, rice and grapevine. Arabidopsis data were obtained from TAIR, whereas poplar and rice data were deduced by consulting the expansin center site (https://homes.bio.psu.edu/expansins/index.htm).
(TIF)

Figure S3 Phylogenetic tree of grapevine expansins together with a set of sequences obtained from Arabidopsis, rice and poplar that represent all of the monocot-eudicot clades present in all the three species proposed [45]. The sequences were aligned with CLUSTALW and a neighbor-joining tree was constructed with MEGA v4.0. Clades are indicated on the right with black bars, numbered using the conventional nomenclature [45].
(TIF)

Figure S4 Expression of expansin genes in individual organs. The expression of expansin genes was considered separately in (A) bud, (B) leaf and (C)rachis. Genes were variance-filtered eliminating 50% of the expansin genes showing low variation between samples.
(TIF)

Table S1 List of real time RT PCR primers.
(DOCX)
