# Supplementary figures and images for: Genome-Wide Analysis of the Expansin Gene Superfamily Reveals Grapevine-Specific Structural and Functional Characteristics
Source: PLoS One. 2013 Apr 16;8(4):e62206. doi: 10.1371/journal.pone.0062206 (PMC3628503; doi:10.1371/journal.pone.0062206)

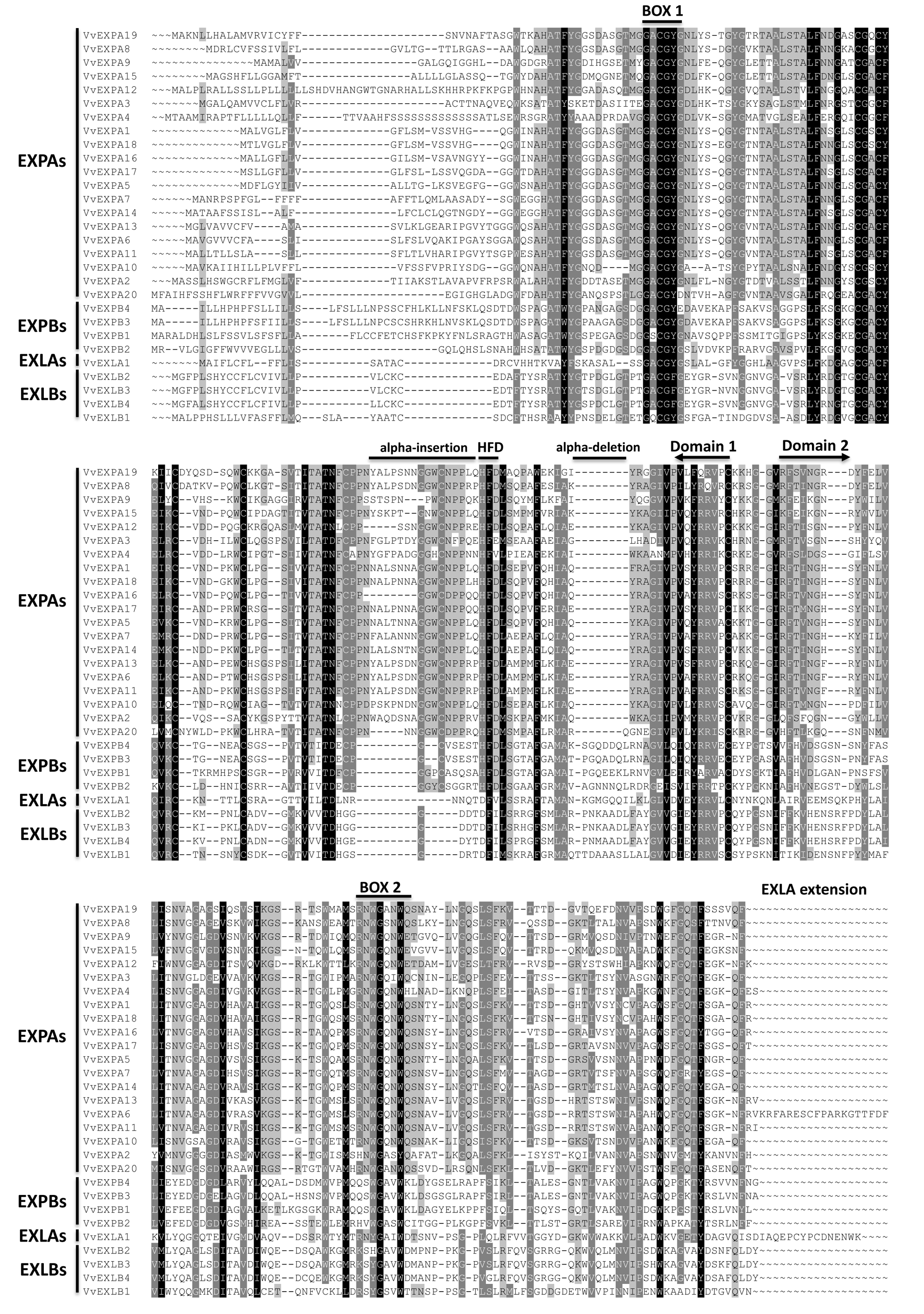

Supplement: Figure S1 — Alignment of grapevine expansin protein sequences. Alignment of all grapevine expansin deduced protein sequences representative of the VvEXPA, VvEXPB, VvEXLA and VvEXLB families. The alignment was created using MAFFT v6.0 and edited with GeneDoc. The family-specific conserved motifs and active sites are indicated. (TIF) [file pone.0062206.s001.tif]

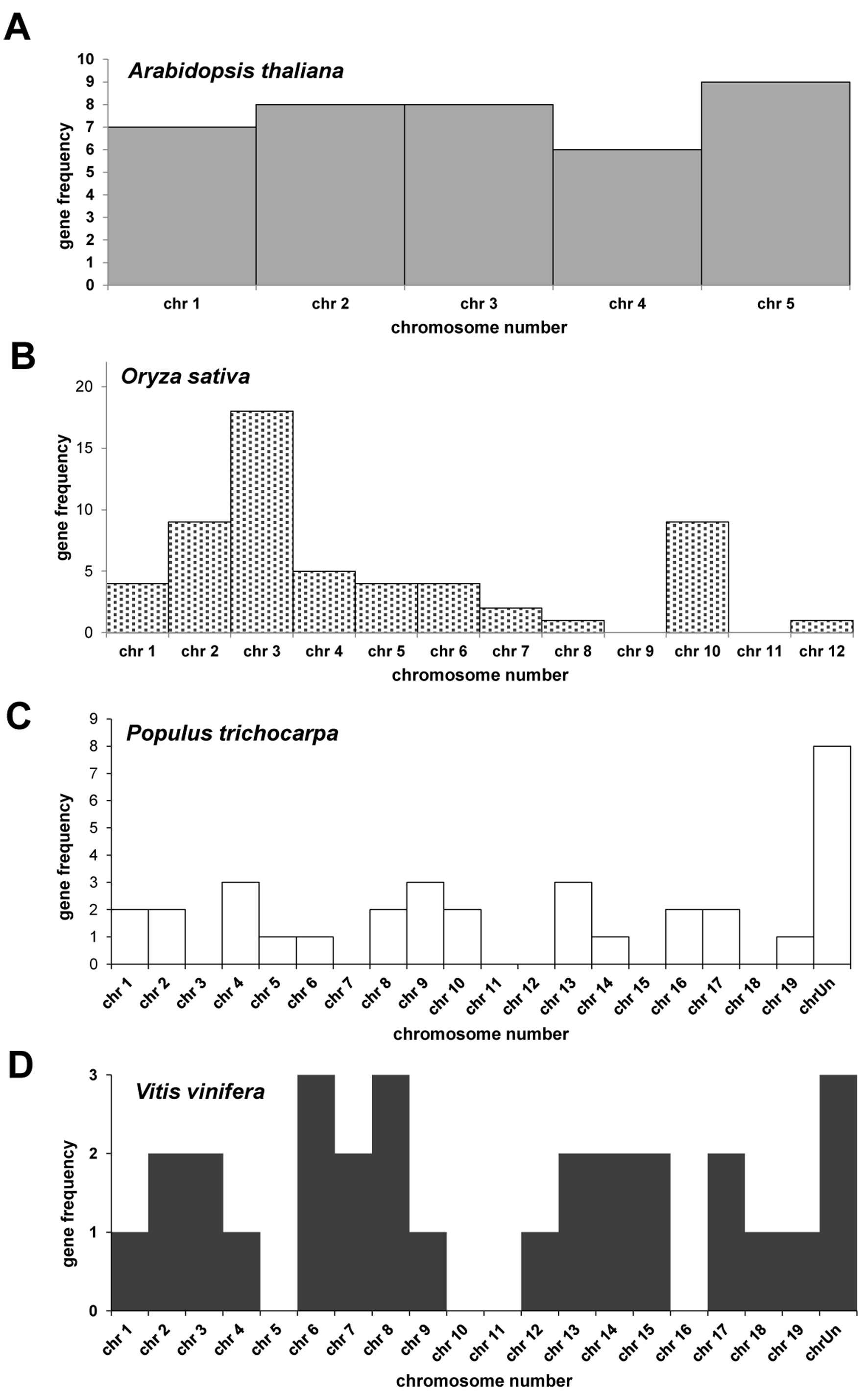

Supplement: Figure S2 — Gene frequency per chromosome of expansins in Arabidopsis, poplar, rice and grapevine. Arabidopsis data were obtained from TAIR, whereas poplar and rice data were deduced by consulting the expansin center site (https://homes.bio.psu.edu/expansins/index.htm). (TIF) [file pone.0062206.s002.tif]

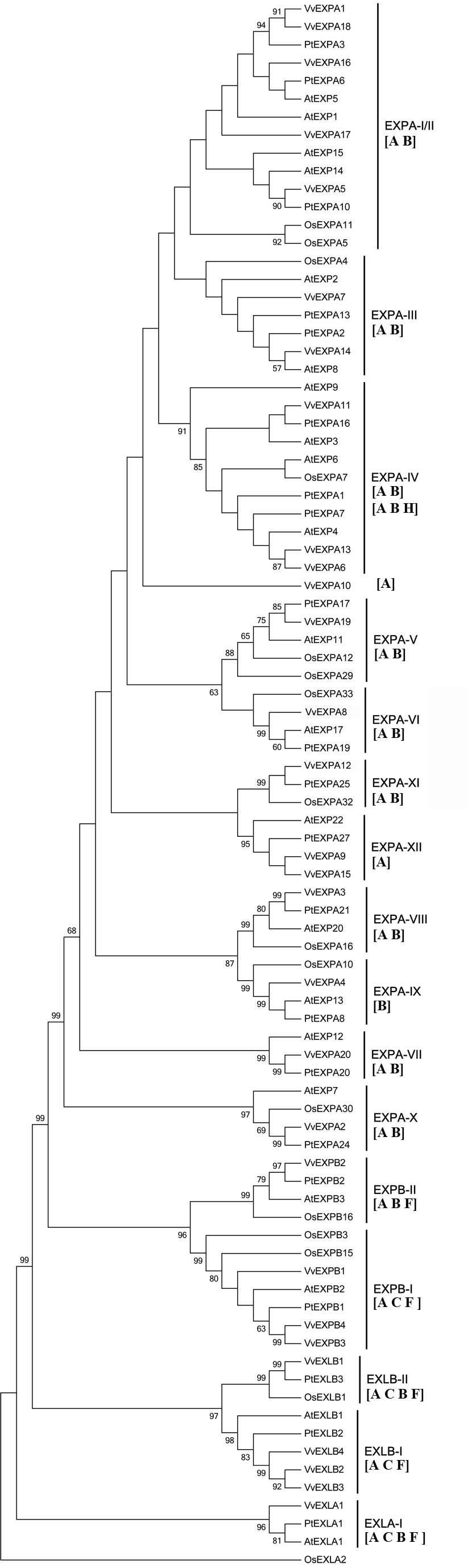

Supplement: Figure S3 — Phylogenetic tree of grapevine expansins together with a set of sequences obtained from Arabidopsis, rice and poplar that represent all of the monocot-eudicot clades present in all the three species proposed [45]. The sequences were aligned with CLUSTALW and a neighbor-joining tree was constructed with MEGA v4.0. Clades are indicated on the right with black bars, numbered using the conventional nomenclature [45]. (TIF) [file pone.0062206.s003.tif]

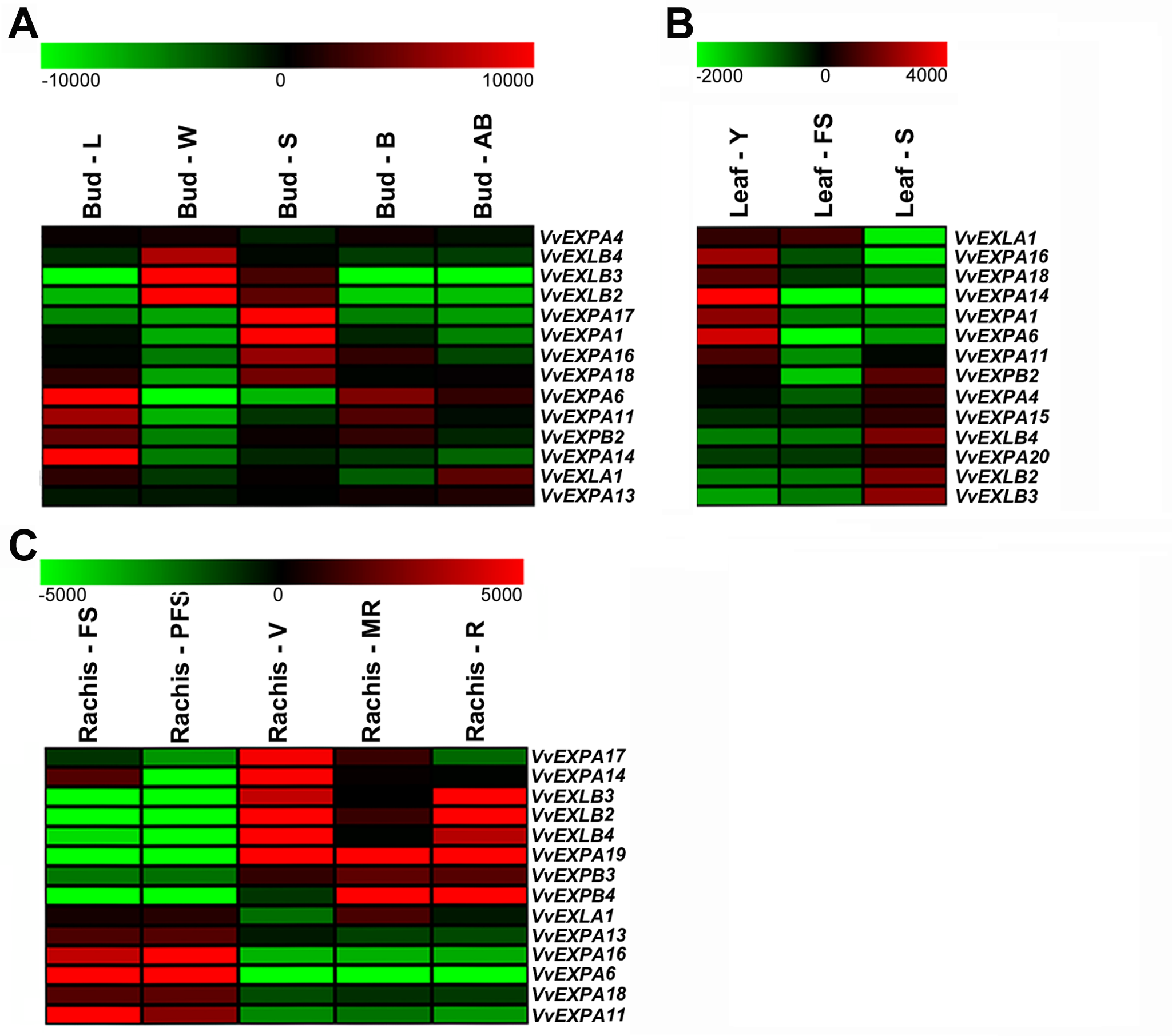

Supplement: Figure S4 — Expression of expansin genes in individual organs. The expression of expansin genes was considered separately in (A) bud, (B) leaf and (C)rachis. Genes were variance-filtered eliminating 50% of the expansin genes showing low variation between samples. (TIF) [file pone.0062206.s004.tif]
