# Supplementary material for: Genome-Wide Analysis of the Expansin Gene Superfamily Reveals Grapevine-Specific Structural and Functional Characteristics
Source: PLoS One. 2013 Apr 16;8(4):e62206. doi: 10.1371/journal.pone.0062206 (PMC3628503; doi:10.1371/journal.pone.0062206)
Supplement: Table S1 — List of real time RT PCR primers. (DOCX) [file pone.0062206.s005.docx]

| Gene ID | PN40024  12X V1 ID | Forward primer (5’ to 3’) | Reverse Primer (5’ to 3’) |
| --- | --- | --- | --- |
| ***EXPA*** |  |  |  |
| *VvEXPA5* | VIT_06s0004g00070 | CAAGCAATGTCTCGCAACT | GCCACATTGTTCGACACTAC |
| *VvEXPA8* | VIT_07s0005g02310 | TGCTGGAGAAGTCTCTAAGG | GCTCTGGCCGTTCAGGTATT |
| *VvEXPA9* | VIT_07s0005g04850 | TAGGATGGTGCAGTCGGATA | GGCAACACAACAAGTACATC |
| *VvEXPA12* | VIT_08s0007g04680 | CTCTACCTCCTGGCATATCG | TCCTTTGTGCCTGGCAGAAC |
| *VvEXPA14* | VIT_13s0067g02930 | GCTAATTGGCACCCGCATG | ATCTAAAAAGCTTGGAGGGC |
| *VvEXPA16* | VIT_14s0108g01020 | AAAGCCCCTGCCACTGCTGA | GACCTCACATAGACTAAGCA |
| *VvEXPA17* | VIT_17s0000g06360 | CAAACTTGCTTAATGGTGTATC | CCAGAATCTACACTAAACTAAC |
| *VvEXPA19* | VIT_18s0001g01130 | CCTTCTCTAGCTCAGTACAG | GGGCGGGCTGCTTCATTAAA |
| ***EXLA*** |  |  |  |
| *VvEXLA1* | VIT_03s0038g03430 | AGGGACTTGCTGTTATTTATG | CTCAAATAATCTAATGCTCTGC |
| ***EXLB*** |  |  |  |
| *VvEXLB3* | VIT_00s0309g00090 | ATACCAAAACTTTGTGCAGATG | CCAACATTGAGAATCCGTGCC |
| *VvEXLB4* | VIT_00s1455g00010 | GTACCAAACCTTTGTGCAGATAA | CCAACATTGAGAATCCGCGC |
| ***UBIQUITIN*** |  |  |  |
| *VvUBQA-52* | VIT_16s0098g01190 | aggcgtgcataacatttgcg | TCTGAGGCTTCGTGGTGGTA |
